# Historically low mitochondrial DNA diversity in koalas (*Phascolarctos cinereus*)

**DOI:** 10.1186/1471-2156-13-92

**Published:** 2012-10-24

**Authors:** Kyriakos Tsangaras, María C Ávila-Arcos, Yasuko Ishida, Kristofer M Helgen, Alfred L Roca, Alex D Greenwood

**Affiliations:** 1 Leibniz-Institute for Zoo and Wildlife Research, Berlin, 10315, Germany; 2 GeoGenetics, Natural History Museum of Denmark, University of Copenhagen, Østervoldgade 5-7, Copenhagen, DK, 1350, Denmark; 3 Department of Animal Sciences, University of Illinois at Urbana-Champaign, Urbana, IL, 61801, USA; 4 Smithsonian Institution, National Museum of Natural History, Washington, DC, 20560-0108, USA

## Abstract

**Background:**

The koala (*Phascolarctos cinereus*) is an arboreal marsupial that was historically widespread across eastern Australia until the end of the 19^th^ century when it suffered a steep population decline. Hunting for the fur trade, habitat conversion, and disease contributed to a precipitous reduction in koala population size during the late 1800s and early 1900s. To examine the effects of these reductions in population size on koala genetic diversity, we sequenced part of the hypervariable region of mitochondrial DNA (mtDNA) in koala museum specimens collected in the 19^th^ and 20^th^ centuries, hypothesizing that the historical samples would exhibit greater genetic diversity.

**Results:**

The mtDNA haplotypes present in historical museum samples were identical to haplotypes found in modern koala populations, and no novel haplotypes were detected. Rarefaction analyses suggested that the mtDNA genetic diversity present in the museum samples was similar to that of modern koalas.

**Conclusions:**

Low mtDNA diversity may have been present in koala populations prior to recent population declines. When considering management strategies, low genetic diversity of the mtDNA hypervariable region may not indicate recent inbreeding or founder events but may reflect an older historical pattern for koalas.

## Background

Though distributed widely across eastern Australia, the koala (*Phascolarctos cinereus*) was apparently a rare animal at the time of the first European settlement of Australia. Many of the relatively large mammals of eastern Australia (such as kangaroos, possums, quolls, and the dingo) were first documented by Europeans almost immediately after the settlement of modern-day Sydney in 1788, but the first European sighting of a koala was not reported until 1798. The first live specimen was secured and brought to Sydney in 1803, and very few detailed accounts of the animal appeared in the decade that followed. Remarkably, it was not until 1816, nearly three decades after the arrival of Britain’s First Fleet to Botany Bay, that the koala was studied in sufficient detail to be given its original scientific name, *Lipurus cinereus*[1]. Most students of the koala have attributed the rarity of the animal in the late eighteenth and nineteenth centuries to the impacts of hunting by Aboriginal Australians. John Gould, among the most notable nineteenth-century naturalists working in Australia, discussed the rarity of the koala and the impacts of Aboriginal hunting upon it, and speculated that it was likely to decline to extinction. Instead, hunting pressure diminished during much of the 19^th^ century, and koala numbers are believed to have risen drastically, such that the koala was apparently a common animal by the middle to late 1800s across much of eastern Australia [1].

Soon after their numbers increased, koala fur became a highly valued commodity, and the koala was hunted with tremendous intensity for the international fur trade. Throughout the late 1800s and early 1900s, millions of koalas are thought to have been killed for their fur every year. Koala declines from intensive hunting during this period were also exacerbated by habitat conversion leading to habitat loss and range fragmentation (the great majority of relevant koala habitat present at European settlement had been cleared by the twentieth century) [1]. Many historical reports also describe mass declines of koalas from various epidemic diseases, dating back to the 1880s [1]. By the 1930s, from the combined effects of hunting, habitat conversion, and disease, koala populations had been extirpated across much of southern Australia and were greatly diminished in the northern range of the species [2,3].

A severe decrease in population size has been shown to lead to loss of genetic diversity in other species [4]. For example, the US grey wolf (*Canis lupus*) lost diversity due to widespread extirpation of local populations. Leonard et al. [5] demonstrated using modern and museum samples that the museum wolf samples had twice the diversity of modern conspecifics. The decrease in numbers of koalas would likewise suggest that the species may exhibit reduced genetic diversity due to population bottlenecks. Reduced genetic diversity is associated with a variety of potential threats in affected populations, such as a decrease in population fitness due to inbreeding, expression of deleterious recessive alleles, reduced disease resistance, and lack of heterosis [2,6].

Several molecular markers have been used to examine the genetic variability of modern Australian koala populations; these have shown that genetic diversity within and gene flow between koala populations is low [3,7-9]. Houlden et al. [10] examined 860 bp of mtDNA hypervariable region in 208 koalas from 16 populations throughout Australia. Koala mtDNA haplotype diversity within populations averaged H= 0.18, which was only one-fifth the level of diversity detected in an Australian omnivore, the greater bilby (*Macrotis lagotis*). Genetic differentiation among the koala populations of Queensland and New South Wales was found to be high, while koala populations from Victoria were little differentiated [10], presumably due to their recent descent from few individuals from the French and Philip Islands. Fowler et al. [11] examined 670 bp of the same mtDNA hypervariable region studied by Houlden et al. [10] from 96 koalas in five southeastern Queensland populations. Haplotype diversity within populations was estimated as H= 0.37, higher than Houlden et al. [10] but still much lower than bilby diversity.

The historical DNA diversity present among koalas can be examined using museum specimens, as mtDNA analyses from museum samples have been reported from many species including Australian wildlife [12-14]. We hypothesized that, compared to modern koalas, there would be greater genetic diversity in the museum specimens, particularly those collected in the 1800s and early 1900s, before (or at the beginning of) the most recent prolonged historical population decline. Using ancient DNA approaches, the diversity of historical haplotypes in the museum koalas was compared to the diversity present in modern koalas.

## Results

**Figure 1:**
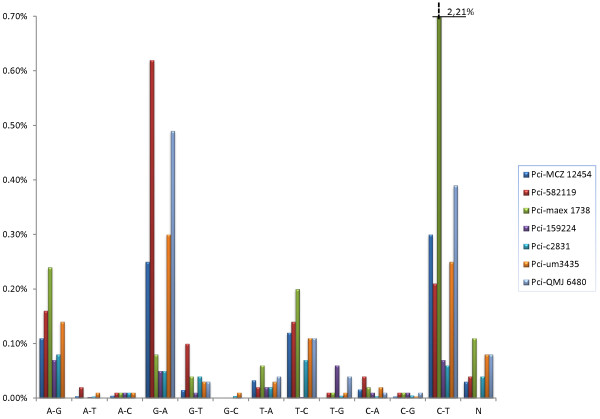
**DNA damage profiles for GS FLX sequenced amplicons of mtDNA from koala museum specimens.** For each koala specimen, the consensus sequence was determined by aligning 454 reads for the individual. Observed differences from the consensus character states were tallied at each nucleotide position for each of the 3 possible non-consensus bases. The total for each possible type of base error relative to the consensus koala sequence was calculated by considering each type of error across all of the nucleotide sites that had the same consensus character state; thus C-T error represents for all sites at which the consensus sequence was cytosine the average proportion of times at which a thymine was present. The percentage for each possible error for each koala is shown. The scale of the *y*-axis was set to a maximum of 0.7%. The single bar interrupted by hatch marks indicates a value of 2.21% for C-T errors in koala Pci-maex1738.

The koala has an 860 bp hypervariable mtDNA region that is adenine and thymine rich. Primer sets were designed to amplify fragments of 73 bp and 112 bp (excluding primer lengths) of this mtDNA region and were tested on a modern koala DNA dilution series. The primers were designed to target small amplicons to accommodate the potential degradation that has often been observed in archival museum samples. We attempted DNA extractions and PCR on 29 different museum samples originally collected from the 1870s to the 1980s (Table 1). Across the 29 samples, we were able to amplify and sequence both of the mtDNA fragments from 15 samples representing 14 individual koalas (Table 1). An additional 1 sample was successfully amplified and sequenced for only the 73 bp mtDNA fragment, potentially indicating that the DNA from that sample was more highly degraded. Thirteen samples failed to amplify for both of the mtDNA fragments. The amplicons were sequenced using two approaches: GS FLX and Sanger direct sequencing (see Materials and Methods for details). The seven GS FLX analyzed samples displayed minimal levels of the damage usually associated with ancient DNA (Figure 1) and the results were found to be comparable between the two approaches. Thus, direct Sanger sequencing of amplicons appeared to be an appropriate strategy for these museum samples.

**Table 1:** Koala samples and sequencing information

**Koala sample origins**	**Designation/ museum no.**	**Collection year**	**Amplified mtDNA**	**Haplotype**	**Sequencing method**
**Northern Australian Samples**					
Bohusläns Museum	Pci-um3435	1891	⊠	K3	GS FLX/ Sanger
Goteborg Museum	Pci-collan18193	1870 - 1891	□		
	Pci-maex1738	1870 - 1891	⊠	K5	GS FLX
Kansas University Museum	Pci-159224	1980s	⊠	K4	GS FLX
Museum of Comparative Zoology	Pci-MCZ 12454*	1904	⊠	K4	GS FLX/ Sanger
	Pci-MCZ 8574*	1904	⊠		GS FLX
Museum of Victoria	Pci-c2831	1923	⊠	K4	GS FLX/ Sanger
	Pci-c2832	1923	□		
Queensland Museum	Pci-QM J6480	1938	⊠	K5	GS FLX/ Sanger
	Pci-QM J2377	1915	□		
	Pci-QM J7209	1945	⊠	K5	Sanger
	Pci-QM J8353	1952	□		
	Pci-QM JM1875	1960s	⊠	K4	Sanger
	Pci-QM JM64	1973	⊠	K4	Sanger
	Pci-424	1970 - 1980s	⊠	K5	Sanger
	Pci-6121^a^	1970 - 1980s	⊠	K5	Sanger
	Pci-7463	1970 - 1980s	⊠	K5	Sanger
	Pci-7625	1970 - 1980s	⊠	K5	Sanger
Royal Ontario Museum	Pci-9111010180	1891	□		
Stockholm Museum	Pci-582119	1911	⊠	K4	GS FLX
U of Michigan Museum of Zoology	Pci-122553	1966	□		
	Pci-124673	1977	□		
**Southern Australian Samples**					
***New South Wales***					
Australian Museum	Pci-AM M17311	1883	□		
	Pci-AM M17299	1883	□		
	Pci-AM M17300	1883	□		
	Pci-AM B4593	1884	□		
	Pci-AM M1461	1899	□		
	Pci-AM M12482	1971	⊠	K1	Sanger
***Victoria***					
Australian Museum	Pci-AM M4841	1930	□		

x Indicates successful amplification and sequencing of sample mtDNA.

* Indicates that the two samples represent the same individual.

^a^ Amplification and sequencing of a single fragment.

**Figure 2:**
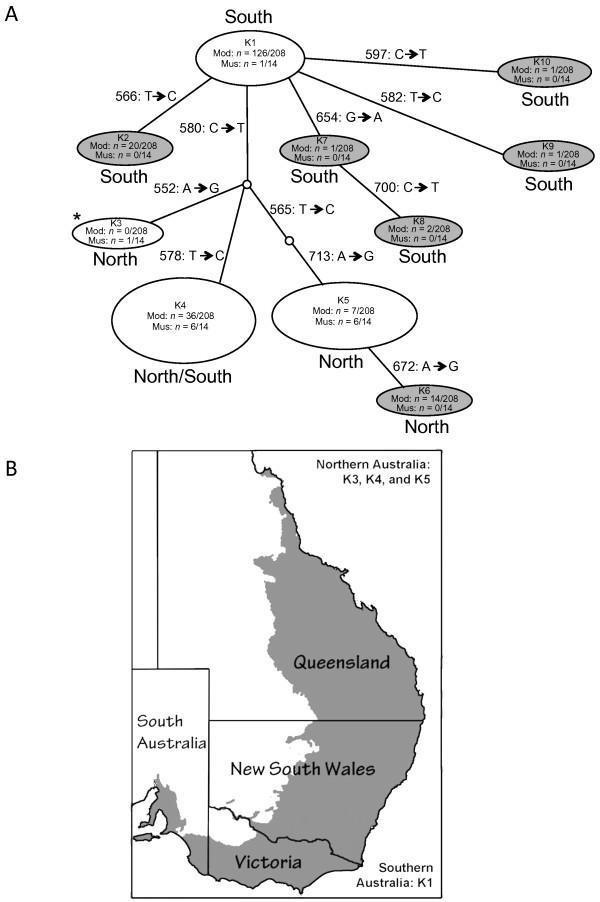
**Panel A shows a minimum spanning network indicating the number of nucleotide differences detected among koala mtDNA hypervariable region haplotypes.** Identical sequences were grouped together as a single unique haplotype. Unshaded nodes indicate haplotype sequences found in both museum and modern samples. Shaded nodes represent sequences detected only in modern samples. The position of the changes relative to the koala reference mtDNA genome (AJ005852) is indicated for each variant site by an arrow e.g. K5 differs from K6 by an A-G change at position 672 where K5 has the reference base adenine and K6 has a change to guanine at that position. Numbers of samples in which each haplotype was detected are indicated for modern (“Mod”) and museum (“Mus”) samples. For haplotype K3 (asterisk), identified by Fowler et al. [11], the number of modern koalas carrying the haplotype was not reported. The geographic distribution of each haplotype (northern or southern Australia, or both) is indicated. Panel **B** shows the approximate current range of koalas (shaded) in Australia. The historical haplotypes identified are indicated inside the parentheses. The map image was adapted from map images provided by the Australian Government, www.ga.gov.au.

The sequences from the two adjacent mtDNA segments indicated that 4 unique haplotypes were present across the 14 koalas (Table 1, Figure 2A). Each of the four museum sample mtDNA haplotypes was identical to one of the previously reported modern haplotypes identified by Houlden et al. [10], and Fowler et al. [11]. No novel mtDNA haplotype was detected among the historical samples.

Minimum spanning networks were generated to demonstrate the relationships across the mtDNA haplotypes (Figure 2A). The koala archival samples carried haplotypes identical to modern haplotypes K1, K3, K4, and K5. Only one or few mutations separated these haplotypes [8,10,11]. All but one of the sequenced museum samples were collected in northern Australia. The haplotypes observed among northern Australian museum samples (K3, K4 and K5) were also found among modern samples collected in northern Australia only (K3, K5), or were found in both northern and southern modern koalas (K4) (Figure 2). Only one of the museum samples from southern Australia was successfully sequenced. The haplotype for this koala (Pci-AM M12482; Table 1) was identical to a haplotype characteristic of modern southern koalas (haplotype K1, Figure 2). Thus for the koala museum specimens, the geographic distribution of haplotypes proved consistent with those of modern koalas.

**Figure 3:**
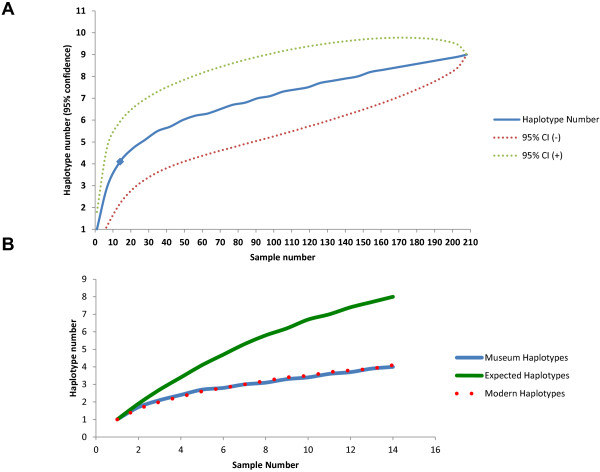
**Rarefaction analyses of modern and ancient koala mtDNA haplotypes.** Panel **A** shows the expected number of haplotypes based on random sampling of the modern koala dataset. Based on the Houlden et al. [8] dataset, one would expect that a random sample of 14 modern koalas would carry 4 unique haplotypes. The set of 14 museum samples for which mtDNA could be sequenced also carried 4 unique haplotypes (blue diamond), completely consistent with expectations based on modern mtDNA diversity. Panel **B** models mtDNA haplotype data based on random sampling of modern koalas, ancient koalas and a conjectural dataset. The solid blue curve represents the rarefied number of mtDNA haplotypes for modern koalas, showing the number of unique mtDNA haplotypes expected given sample sizes of up to 14. The overlapping red dotted line indicates the number of mtDNA haplotypes for museum koalas. The overlap between curves representing modern and ancient koalas indicates that koala mtDNA diversity was not substantially higher in the museum samples than the modern samples. The green solid line represents rarefaction analysis of a conjectural population with twice the number of mtDNA haplotypes as modern koalas. The curve for the conjectural dataset deviates substantially from the curves drawn from modern and museum data, even at small sample sizes.

The number of museum sample sequences that we were able to obtain was lower than the number of modern samples that have previously been sequenced. Rarefaction analysis was used to estimate expected haplotype diversity of a modern sample set of the same size as the museum sample set. Using the modern DNA data, rarefaction analysis suggested that if 14 modern koalas had been sampled at random, 4 haplotypes would be identified, the same number of haplotypes that we were able to identify from our 14 museum samples (Figure 3A). This suggested that ancient koalas carried mtDNA diversity similar to that present in modern koala populations. Rarefaction analysis of the modern sample set was also plotted to evaluate whether the sequencing of additional museum specimens would be likely to identify a larger number of mtDNA haplotypes (Figure 3A). The analysis suggested that even a 2-fold increase in museum samples would have identified only a single additional haplotype (Figure 3A). An additional analysis, separating the 7 oldest samples and seven youngest into two sets, predicted no differences in the expected number of retrievable haplotypes between the two sets (not shown).

Rarefaction analysis was also performed using a conjectural dataset in which the number of haplotypes of mtDNA was twice that actually observed e.g., 8 rather than the actual 4 haplotypes present across 14 samples. This conjectural dataset was meant to illustrate the initial hypothesis that mtDNA diversity would be higher in museum than in modern samples, as had been found in the greater bilby and grey wolf [5]. The results are shown in Figure 3B. As koala sample size increases, the number of haplotypes increases for modern and museum specimens, with the curve based on actual museum specimen results following the same trajectory as the curve followed by modern samples. By contrast, the conjectural diversity curve, postulating twice the diversity in the past relative to the present, had a different trajectory, with even a sample set comprised of a much smaller number of koalas yielding more haplotypes than actually observed (Figure 3B). The conjectural curve follows a much higher trajectory than actual modern or museum data sets. Thus the low number of historical haplotypes likely reflects limited haplotype diversity in the museum koalas equivalent to that in the modern samples, rather than the effects of limited sampling.

## Discussion

Our study represents the first analysis of koala mtDNA to incorporate museum specimens. Sixteen of twenty-nine samples amplified for mtDNA; these ranged in year of collection from the late 1800s to the 1980s. Previous surveys of koalas have revealed that mtDNA diversity is quite low in modern populations [11]. We hypothesized that there would be greater diversity in the museum samples, particularly those from the 1800s and early 1900s, as compared to modern samples, expecting that genetic diversity would be greater prior to the severe population reductions that occurred from the late 1890s through the early 20^th^ century. Sequencing of 201 bp of the mtDNA hypervariable region from 14 koalas yielded four haplotypes, all of which were present in modern koalas (Table 1 and Figure 2). The lack of novel mtDNA haplotypes in archival northern Australian specimens collected from a variety of populations and time points suggested that limited mtDNA variation was present in northern koalas before the known historical population reduction, which lowered koala numbers in northern Australia from approximately 1 million animals to ten thousand by 1927 [11].

Koala populations declined drastically across Australia in the early 20^th^ century, yet our museum sample data did not reveal that diversity was higher before the bottleneck. Rarefaction analysis indicated that these results were not due to unequal modern and museum sample sizes (Figure 3). Doubling the museum sample size can be predicted to have yielded only one additional unique haplotype. If museum koalas had greater mtDNA diversity, then more haplotypes should have been detected in the museum sequences, as occurred in ancient DNA studies of other species such as the US grey wolf [5]. However, this was not the case and we concluded that our results are not an artifact of the small sample sizes.

This provides an indication that older events may have been more important in shaping modern genetic diversity in koalas. Late Pleistocene extinctions in Australia, attributed to the impacts of Aboriginal arrival, climate change, or their combination, resulted in the extinction of most of the continent’s larger animals (megafaunal marsupials, birds, and reptiles), as well as the extinction or decline of some medium-sized species, including koalas [15]. Prior to this extinction event, two koala species (both the modern *P. cinereus* and the larger *P. stirtoni*) co-existed in Australia, and the modern koala had a wider distribution that extended to south-western Western Australia [16,17]. The extinction of one koala species and the reduction of the geographic range of the living koala since the Pleistocene provide strong indications of Late Pleistocene koala declines, which in addition to reducing the taxonomic diversity of koalas, would also have likely impacted the genetic diversity of *P. cinereus*. Further, if the rarity of koalas at the time of European arrival to Australia provides an indication of a historical baseline for koala abundance under the impacts of Aboriginal hunting, it can be hypothesized that the limited genetic diversity seen in modern koalas might reflect a long history of relatively low population numbers throughout the range of koalas during the Late Pleistocene and Holocene, another demographic factor that may have strongly limited koala genetic diversity in recent millennia.

Historically low mitochondrial diversity is not unique to Australian koalas. Among Australian marsupials, low mtDNA diversity has also been reported in the Tasmanian devil (*Sarcophillus harrisii*). A recent study by Miller et al. [14] that compared modern Tasmanian devil mtDNA diversity with historical diversity based on 175 modern samples and six museum specimens collected between 1870 and 1910 revealed five historical haplogroups. Four were identical to haplogroups in modern Tasmanian devils and only one was unique to the historical samples. The authors concluded that Tasmanian devil mitochondrial diversity has generally been low over the past century even though the population had started to decline more recently due to disease [18]. Similarly, the extinct thylacine (*Thylacinus cynocephalus*) demonstrated low genetic diversity, which may have been a consequence of the separation of Tasmania from mainland Australia [19,20]. In non-Australian fauna, the Iberian lynx (*Lynx pardinus*) has maintained extremely low genetic diversity for at least 50,000 years [21]. Thus, low historical genetic diversity is not unusual among species for which ancient and historical samples have been analyzed. Nonetheless, the low koala mtDNA diversity does contrast with those of another Australian marsupial that went through a bottleneck, the northern hairy-nosed wombat. This wombat species lost half its diversity through a population bottleneck that was concurrent with the bottleneck that affected koalas; although the wombat went through a much greater proportional decline in population than the koala [22]. Thus, the continously low genetic diversity detected among koalas, Tasmanian devils and the Iberian lynx is not typical for all species. Some species examined across very long periods of time have demonstrated loss of genetic diversity as population sizes decreased, for example the Beringian steppe bison; while others tended to maintain their genetic diversity and population sizes across time, for example the woolly rhinoceros [23,24].

Koalas may have experienced population expansions and subsequent crashes repeatedly throughout the Quaternary, depending on the availability of eucalyptus, and, after human arrival, the intensity of human hunting. These more ancient bottlenecks may be responsible for the low diversity evident in our museum samples [17]. Koalas are currently threatened by co-infection with two lethal pathogens that are putting substantial pressure on the host species. The koala retrovirus (KoRV) is an oncogenic retrovirus that can cause high levels of mortality in captive and wild koala populations [25,26]. High titres of KoRV are correlated with infection by *Chlamydia*[27], which is now a major pathogen of wild koalas. Lack of genetic diversity may result in a general inability of the koala to resist either pathogen. Thus, biodiversity management strategies may have to consider prioritizing the preservation of KoRV-free populations given that genetic diversity may be low across the species.

The use of museum samples for genetic studies is not without difficulties. Besides the potential for a high failure rate, which proved to be over 50% in this study, the generation of error-free sequence is a crucial issue. Criteria have been suggested for authentication of ancient DNA sequences [28]. One of the suggested criteria is sub-cloning and sequencing of multiple clones. However, when a single sequence variant is expected, as with mtDNA haplotypes, it has recently been suggested that direct sequencing may be as accurate as cloning and sequencing [29]. We compared the results of high throughput sequencing of PCR amplicons to those of direct Sanger sequencing of PCR products. The high throughput sequencing data suggested that DNA damage affected only ca. 1% of the sites. Presumably Taq polymerase errors or nuclear copies of mtDNA (numts) also contributed to the sequence errors, although the effects of any numts could not be separately quantified [30]. Nonetheless, the consensus sequence of the reads was identical to the direct consensus Sanger sequencing results. Thus, under some circumstances, direct sequencing of ancient DNA may be adequate if steps are taken to confirm that reliable sequences can be obtained.

## Conclusions

Koalas appear to have been characterized by low mtDNA diversity for at least the last 120 years. Contrary to expectations, historical diversity of the koala did not appear to differ from modern diversity and thus the most recent episode of overhunting likely played less of a role in the current paucity of mtDNA diversity than more ancient factors. Koala genetic diversity may have been affected by ancient climatic changes affecting their range or by previous aboriginal hunting pressure, before being impacted more recently by European Australian exploitation. The combined pressure on koalas has likely limited the accumulation of genetic diversity. When considering management strategies, it may be noted that low genetic diversity may not indicate recent inbreeding or founder events but may reflect a historical pattern for koalas. However, the long-term low diversity may have implications for the koala’s ability to resist disease, as may be apparent from the spread of koala retrovirus and associated *Chlamydia* infections.

## Materials and methods

### Samples

Koala museum skin samples were generously provided by the Bohusläns Museum, Goteborg Museum, Kansas University of Kansas Natural History Museum, Museum of Comparative Zoology, Museum of Victoria, Queensland Museum, Royal Ontario Museum, Stockholm Museum, University of Michigan Museum of Zoology, and the Australian Museum (Table 1). Twenty-nine specimens from 28 individuals dating from the early 1870s to the early 1980s were sampled. When collection dates could not be constrained to a specific year, the range of dates possible for collection is listed.

### DNA extraction

All extractions were performed in a dedicated ancient DNA facility in plexiglass PCR hoods, one hood dedicated to DNA extraction and a second hood to PCR setup. The room was never used for cell, molecular or genetic work on modern samples, and the Wildlife Diseases Department of the Leibniz-Institute for Zoo and Wildlife Research had never worked with koalas prior to the project. Personnel wore protective clothing to ensure that samples were not contaminated by the researchers during extraction or PCR setup. Approximately 250 mg of skin tissue was extracted using the Geneclean Ancient DNA Extraction Kit from MP Biomedicals, USA. The protocol of the kit was followed according to the manufacturer’s instructions; the same kit had previously been used successfully for ancient DNA extraction from samples of rat skin and woolly mammoth bone [31,32]. Mock extractions were performed with each set of koala museum specimens as a control for contamination during the extraction procedure. Subsequent to each extraction, the DNA was further purified using QiaQuick spin columns (Qiagen) as described in Gilbert et al. [33].

### PCR and sequencing

Primers were designed based on conserved regions in modern koala mtDNA. Primers targeted regions of the mitochondrial DNA control region found to have high variability in koalas according to Houlden et al. [10]. The primers were tested at the University of Illinois on modern koala DNA. Combinations tested were PCI-CRs1-F1 (5’- AGTACATTCATTTATTTACCACTAGCAT-3’) or PCI-CRs1-F2 (5’-ATAGTACATTCATTTATTTACCACTAGCA-3’) with PCI-CRs1-R1 (5’- AAGATGATTTATATGTAATTCTAGATACGC-3’) to amplify a ca. 73 bp target (exclusive of primer lengths). PCI-CRs2-F1 (5-ACCAAATGCGTATCTAGAATTACA-3’) was combined with PCI-CRs2-R1 (5’-TTTGCTTGGATTGGATATGCT-3’) and PCI-CRs2-F2 (5’-TACCAAATGCGTATCTAGAATTACA-3’) with PCI-CRs2-R2 (5’-TTGCTTGGATTGGATATGCT-3’). Primer sets that amplified short mtDNA regions were used in order to minimize negative results associated with DNA damage in museum samples. Both PCI-CRs2 primer combinations target the same 112 bp (without primers) region. The two reverse primers of the PCI-CRs1 amplicon overlaps with the two forward primers of the PCI-CRs2 amplicon and thus, the region sequenced did not overlap. A combination of the two sets was also used (PCI-CRs1-F1/PCI-CRs2-R1) to obtain a larger product (~201 bp) encompassing both the 73 and 112 bp amplicons including the region of primer overlap between the smaller amplicons. The primers were tested with a dilution series of koala DNA to mimic the scarcity of DNA present in ancient samples. Each of the selected primers and combinations except PCI-CRs1-F1/ PCI-CRs2-R1 worked under this test regime on the lowest koala DNA dilution.

For all the samples, multiple independent PCRs were performed for each extract, and multiple extracts were obtained from each specimen. For all samples positive PCRs were replicated at least twice per primer set to avoid overestimation of diversity associated with ancient DNA damage. All amplifications from museum specimens were performed in a total volume of 34 μl using 5.5 μl of sample extract, 200 nM of primers, and 0.5 U Platinum HiFi supermix (Invitrogen). Cycling conditions were: 94°C for 4 min; 60 cycles at 94°C for 30 s, 55°C for 30s, 72°C for 30s; and at 72°C for 10 min, with the samples then held at 4°C [31]. PCR products were visualized on a 3% agarose gel using GelRed nucleic acid gel stain by Biotium. PCR products were purified using the Qiaquick PCR clean up kit (Qiagen).

The positive samples were amplified in duplicate or triplicate to control for DNA damage induced errors that almost universally characterize ancient or degraded DNA samples. A subset of 8 samples from 7 individual koalas, representing the range of collection dates that provided positive results, was selected for GS FLX sequencing using a specific multiplex identifier (MID) according to manufacturer’s instructions (Roche Life Sciences) (Table 1). The sequencing coverage depth obtained using this method effectively substitutes for the laborious cloning steps usually applied in ancient DNA studies and provides equivalent information with much higher sequence coverage [34]. Analyses of the GS FLX sequences for these seven samples indicated that only a very low degree of DNA damage was present (Figure 1). For each individual, every position along the sequences was examined for variation among reads, with the consensus character state (present at a frequency >97% in every case) recorded for each nucleotide site for each individual. The proportion of sequences that had any of the three non-consensus character states was also recorded. Across the length of the sequence, there are 12 types of observable base errors, since for each of the 4 consensus character states there are three potential non-consensus character states, each representing a type of error (Figure 1). The percentage of reads with each type of error was averaged across the length of the sequence; for example the proportion of C to T changes was calculated by examining all positions at which a cytosine was the consensus character state. At all of these positions, the proportion of reads with a thymine was recorded, with the average rate of C-T error estimated by calculating, across all nucleotide sites at which the consensus was cytosine, the proportion of all reads with thymine. For six out of seven koalas, each of the 12 possible base errors comprised less than 1% of the total reads across all nucleotide positions. One koala, Pci-maex1738, had an elevated C-T error rate (Figure 1). However, for this individual, several positions with cytosine had relatively low GS FLX read coverage, and the higher error estimate was due to lower read coverage depth at a few positions. Thus, this was a sequence sampling effect rather than actual increased damage. Even after discounting these artifacts, C-T and G-A transitions were more frequent than other apparent base changes (Figure 1), consistent with the damage profiles expected for ancient DNA [35].

Given that DNA damage induced change appeared to be low across the koala museum specimens, we hypothesized that direct Sanger sequencing would be as effective for sequencing as the GS FLX method. Four of the seven high throughput sequenced koala samples were Sanger sequenced directly (Pci-MCZ 12454, Pci-c2831, Pci-um3435, and Pci-QM J6480) to allow for comparison of the two methods; among them were two of the koalas that showed a higher percentage of damage induced change for certain substitutions (Pci-c2831 and Pci-QM J6480 Figure 1 for example). The direct Sanger method generated sequences identical to those of consensus sequence of GS FLX for all four samples. In two individuals, a single base conflict between the first and second amplicon sequence result was resolved by a third direct sequencing. However, none of the museum koala samples indicated multiple co-occurring sequences or unreadable bases were present. Based on the consistency between direct and high throughput sequencing results, the remaining samples were directly Sanger sequenced. Haplotype sequences are included in the Additional Files 1 and 2.

Of the 3 samples that were successfully amplified with only a single primer set, only Pci-6121 yielded a koala sequence. The haplotype based on this single fragment was not novel. Since only one mtDNA fragment was retrieved from Pci-6121 and it was identical to K5 haplotype it was not further analyzed (Figure 2).

### GS FLX data DNA damage estimation

GS FLX sequence reads were first sorted by MID and .fna and .qual files were transformed into fastq format using perl scripts. Primer regions were removed from the analysis by trimming both ends of each read, and only fragments 36 bp and longer were considered for mapping assembly. Each one of the seven datasets was then mapped to a reference corresponding to the *P. cinereus* mitochondrial control region using BWA (Version 0.5.9) [36]. The following GenBank mitochondrial control region references were used for sequence mapping, i.e., AJ005848, AJ005847, and AJ012063. The reference sequences were chosen based on haplotype similarity. Alignments were then converted to pileup format (samtools 0.1.17) [37] and a perl script was designed to estimate damage by extracting and sorting all the substitutions present at each position in the alignment. The algorithm generated per-position variation and summarized data, which allowed the percentage determination of damage patterns.

### Alignments, minimum spanning networks, and rarefaction analysis

Alignment and comparison of the archival sample data with modern sequences were performed using Clustal X and Bioedit, respectively. The modern koala sequences were obtained from Genbank (accession numbers AJ005846-AJ005863, AJ012057-AJ012064). Minimum spanning networks of the haplotypes were generated using TCS 1.21 software at the default 95% connection limit [38]. Rarefaction analysis was performed using Analytic Rarefaction 2.0 software that uses Tippers [39] equations to estimate the number of haplotypes. As input we used Houldens et al. [8] 208 koala dataset and analysis was performed following the developers instructions [40]. Plotting of the results was performed using Microsoft Excel 2010.

## Competing interests

The authors declare no competing interests.

## Author contributions

KT performed ancient DNA extractions, PCR and sequencing experiments. YI performed all modern koala DNA work. KT and MCAA performed bioinformatics and phylogenetic analyses for the study. KMH provided koala samples and curatorial information and wrote parts of the manuscript. KT, ALR and ADG designed the study, contributed to the experimental work and analysis and wrote the manuscript. All authors read and approved the final manuscript.

## Supplementary Material

Additional file 1 FASTA formatted files of the consensus haplotype sequences for the 73 bp and 112 bp museum koala amplicons. Click here for file

Additional file 2 FASTA formatted files of the consensus haplotype sequences for the 73 bp and 112 bp museum koala amplicons. Click here for file
